# Synthetically designed circRNA can be used to target cardiovascular relevant microRNAs to improve cellular function

**DOI:** 10.1038/s41598-026-46870-7

**Published:** 2026-04-09

**Authors:** Solveig Böttcher, Katrin Kalies, Kai Knöpp, Susanne Koch, Claudia Pilowski, Stefan Hüttelmaier, Daniel Sedding

**Affiliations:** 1 Division of Cardiology, Angiology and Intensive Medical Care, Department of Internal Medicine III, Mid-German Heart Center, University Hospital Halle, Martin-Luther-University Halle-Wittenberg, Ernst-Grube-Strasse 40, 06120 Halle (Saale), Germany; 2 Institute of Molecular Medicine, Section for Molecular Cell Biology, Faculty of Medicine, Martin-Luther-University Halle-Wittenberg, Halle (Saale), Germany

**Keywords:** circRNA, miR-21, miR-146a, Vascular cells, Cardiology, Cell biology, Diseases, Molecular biology

## Abstract

Vascular aging, endothelial dysfunction, and cellular senescence are interconnected processes that drive cardiovascular diseases (CVDs). Several studies have confirmed that microRNAs (miRNAs) are closely associated with the development of CVDs. Among them, miR-21-5p and miR-146a-5p have emerged as critical players in CVDs: MiR-21-5p is implicated in promoting inflammation and fibrosis, while miR-146a-5p influences vascular smooth muscle cell proliferation and inflammatory responses. Targeting these miRNAs for therapeutic intervention could provide a novel strategy to mitigate the deleterious effects of vascular aging. Recent advances in RNA-based therapeutics have led to the exploration of circular RNAs (circRNAs) as stable and effective miRNA inhibitors. Due to their circular structure, circRNAs exhibit greater stability than linear miRNA inhibitors, such as anti-miRs. This study aimed to evaluate the expression of miR-21-5p and miR-146a-5p in senescent human vascular endothelial cells and vascular smooth muscle cells (VSMCs) and to assess whether synthetic circRNAs could effectively inhibit these miRNAs. In a series of experiments, human umbilical vein endothelial cells (HUVECs) and VSMCs were transfected with custom-designed circRNAs targeting these miRNAs. Here, we want to focus on VSMCs. The effects on miRNA expression, target gene regulation, and cellular functions such as proliferation and migration were analyzed. Our findings demonstrate that circRNA-mediated inhibition of miR-21-5p and miR-146a-5p significantly affects gene expression and cellular behavior in senescent vascular cells, suggesting that circRNAs may serve as more efficient and stable therapeutic agents compared to traditional anti-miRs. By addressing these objectives, this study contributes to the growing body of knowledge on circRNA-mediated miRNA inhibition and its potential implications for vascular health. The results from this study not only enhance our understanding of miR-21-5p and miR-146a-5p in vascular senescence but also provide a foundation for future therapeutic strategies.

**Supplementary Information:**

The online version contains supplementary material available at 10.1038/s41598-026-46870-7.

## Introduction

Over the last decade, microRNAs (miRNAs) have emerged as essential post-transcriptional regulators of gene expression in vascular biology^1–3^. By binding to the 3’ untranslated regions (UTRs) of target messenger RNAs (mRNAs), miRNAs control diverse processes such as endothelial function, vascular smooth muscle cell (VSMC) phenotype switching, and inflammatory activation^4–6^. Dysregulated miRNA expression has been implicated in both the initiation and progression of cardiovascular disease^4,7^ with miR-21-5p and miR-146a-5p identified as key modulators in vascular pathology^6,8,9^. miR-21-5p promotes fibrotic remodeling, enhances proliferation and migration of VSMCs, and impairs endothelial cell angiogenesis by targeting tumor suppressor genes such as PTEN, PDCD4, and SPRY1^10–12^. MiR-146a plays a dual role in cardiovascular biology by regulating both inflammation and vascular remodeling. It targets IRAK1 and TRAF6, acting as a negative feedback regulator of the NF-κB pathway and suppressing pro-inflammatory signaling^6,13,14^. Conversely, miR-146a promotes proliferation by targeting KLF4, and inhibits angiogenesis in vascular cells via downregulation of NOTCH1, NOX4, and NRAS^15–17^.

Hence, the therapeutic inhibition of miRNAs represents a promising strategy to counteract vascular pathologies. Antisense oligonucleotides (anti-miRs) have been widely used for miRNA silencing but are limited by their relatively low stability, susceptibility to nucleolytic degradation, and the requirement for chemical modifications to achieve sustained effects *in vivo.* These shortcomings highlight the need for alternative, more robust miRNA inhibitory platforms.

Circular RNAs (circRNAs) have emerged as a structurally and functionally unique class of RNA molecules with significant potential for therapeutic applications, particularly in the context of microRNA (miRNA) inhibition. CircRNAs are characterized by their covalently closed-loop structure formed through a back-splicing mechanism that joins a downstream 5’ splice site to an upstream 3’ splice site. This configuration enhances exonuclease resistance and results in superior stability compared to their linear counterparts^18^. The sponge-like behavior of circRNAs with the sequestration of miRNAs offers a natural blueprint for the design of synthetic circRNAs capable of targeted miRNA inhibition.

Synthetic circRNAs have been engineered to contain multiple tandem binding sites for specific miRNAs (typically 4–8 sites) separated by short linker sequences to facilitate efficient binding without compromising structural stability. Moreover, circularization itself dramatically enhances intracellular half-life, with reported half-lives exceeding 20 h for circular versus ~ 10 h for linear equivalents^19^.

Beyond structural advantages, synthetic circRNAs also offer functional flexibility. They can be delivered as naked RNA, encapsulated within nanoparticles (e.g., PEI-based carriers), or expressed via plasmid-based vectors designed with flanking intronic elements to promote endogenous back-splicing^19–21^. Their modular design allows for high programmability, enabling selective targeting of disease-relevant miRNAs. In cardiovascular research, where miR-21-5p and miR-146a-5p are known to contribute to vascular inflammation and remodeling, synthetic circRNAs provide a compelling approach for precise and sustained gene regulation.

In this study, we focused on developing, producing, and applying synthetic circRNAs to target vascular cells. Based on current literature, we optimized their molecular design and validated their effects in vascular cells. In order to determine whether these processes are universally applicable, circRNA was applied to two different microRNAs in different vascular cells. This work aims to advance the utility of circRNAs as versatile tools for miRNA inhibition in the cardiovascular field and to contribute to the broader landscape of RNA-based therapeutics.

## Materials and methods

Further information can be found in the supplementary material.

### Cell culture

All in vitro experiments were performed on human umbilical vein endothelial cells (HUVECs) and vascular smooth muscle cells (VSMCs) purchased from Lonza. Cells were cultivated in an endothelial or smooth muscle cell growth medium (PromoCell) at 37 °C with 5% CO_2_ in a humidified incubator, grown to confluency, and passaged in a 1:3 ratio. Early replicative senescence was induced by serial passaging as previously described. Senescent status was not defined by passage number alone but was validated using established molecular markers of cellular senescence consistent with commonly accepted approaches for the characterization of vascular cell senescence (Supplementary Fig. 1)^22,23^.

### circRNA design

Circular RNAs were designed and produced as described previously^18,24^. Double-stranded oligos were designed with SnapGene and ordered from Eurofins. The plasmid backbone contains a T7 promoter sequence, a stem-loop, a constant region, and an EcoRI and XbaI restriction site, used to insert the oligos either containing four has-miR-21-5p, has-miR-146a-5p, or cel-miR-239b binding sites (Supplementary Fig. 2). Cel-miR-239b was used as a control sequence. The plasmid region containing the T7 promoter and oligos was amplified with PCR using a sense and antisense primer (Eurofins) and purified using the PureLink Quick Gel Extraction Kit (Invitrogen) according to the manufacturer’s protocol. The DNA template was used for in vitro transcription, which was performed using the HiScribe T7 High Yield RNA Synthesis Kit using triphosphates. DNA templates were digested by DNase I (New England BioLabs). Transcripts were purified using the NEB Monarch RNA Cleanup Kit, followed by dephosphorylation of the 5’-terminal triphosphate resulting from the in vitro transcription reaction using Quick CIP, which must be removed before phosphorylation by the 10x T4 Polynucleotide Kinase. In between and after these steps of de- and rephosphorylation, the products were again purified using the NEB Monarch Cleanup Kit (New England BioLabs). The linear RNA substrates were then used for the circularization process. For the circularization, highly concentrated T4 RNA ligase (New England BioLabs) was used. After a fourth purification step, RNase R exonuclease treatment was used to digest linear transcripts. The circularization was then validated using a 15% polyacrylamide-urea gel. Due to the incomplete digestion of linear transcripts by the RNase R treatment, polyacrylamide gel electrophoresis (PAGE) purification was used to obtain pure circular RNAs. Therefore, instead of the RNase R treatment step, the RNA was applied to a 15% polyacrylamide-urea gel and run at 25 mA for about 20 min. With UV-shadowing, the circular RNA was cut out of the gel and purified using PK buffer.Northern Blot.

For infrared northern blotting of miRNAs and ncRNAs, total RNA was separated in a 15% denaturing TBE–urea gel at 25 mA and transferred for one hour at 30 V onto nylon membranes (Roche) using a system from Biorad. After cross-linking (150 mJ/cm2) and washing the membrane using washing buffer (0.1xSSC, 0.1% SDS), the membrane was incubated at 20 °C overnight in 0.2ng/µl DY782-labeled circRNA (Eurofins) in PerfectHyb Plus hybridization buffer and monitored using an Odyssey Scanner (LI-COR). For normalization, a labeled 5 S probe was used.

### RNA transfection

Transfection of the cells with circRNAs was performed using RNAiMax (Thermo Fisher Scientific) and Opti-MEM (Thermo Fisher Scientific) according to the manufacturer’s protocol. Briefly, circRNAs or anti-miRs were mixed with Lipofectamine RNAiMax and shortly incubated in Opti-MEM media. Cells were seeded in antibiotic-free media and treated with the liposomal complexes. The final concentration of circRNAs was either 2 nM or 5 nM, and the final concentration of anti-miRs was 50 nM. The efficiency of miRNA inhibition was assessed by (qRT)-PCR.

### RNA-isolation, reverse transcription, and qRT-PCR

The miRNeasy Micro Kit (Qiagen) was used for the simultaneous isolation of RNA and miRNA according to the manufacturer’s protocol. Briefly, cells were washed with PBS (3x), lysed, genomic DNA was removed, and total RNA was eluted through a column-based isolation.

RNA was transcribed to cDNA using the High-Capacity cDNA Reverse Transcription Kit (Thermo Fisher Scientific), and qRT-PCR analysis was performed with the Blue S’Green qPCR Kit (Biozym). Both kits were used according to the manufacturer’s instructions with an input of 200 ng total RNA. A list of used primers is shown in the supplementary table (Supplementary Table 1).

### Proliferation and migration

Proliferation was assessed via cell counting in a life-cell imaging approach. Cells were seeded to sub-confluency, and images were taken every 30 min with a life-cell imaging system over 24 h. The number of cells was counted in every brightfield image.

Migration capacity was determined by a scratch-wound assay. The migration assay was performed in 24-well plates, and the cells were seeded to confluency: 30,000 VSMCs or 40,000 ECs per well, with a total medium volume of 600 µl per well. A scratch-wound was done with a pipette tip. Images were taken every 30 min over 24 h with a life-cell imaging system. Scratch area and cell area were determined for every image. All experiments were performed with the Life Cell Imaging System Cytation 1 (Biotek), and image analysis was performed with the software Gen 5 (Biotek).

### Immunofluorescence staining of cells in vitro

For immunofluorescence staining of the cells, the cells were seeded in 8-well chamber slides. After adherence, cells were fixed for 30 min, blocked, and incubated with the primary antibody overnight. On day 2, slides were washed and incubated with the secondary antibody for 2 h. After additional washing steps, the slides were embedded in DAPI, closed with a cover slip, and sealed. A list of used antibodies can be found in the supplemental table (Supplementary Table 1).

### Statistical analysis

Datasets were analyzed using GraphPad Prism 10. All data are represented as mean ± standard deviation; n indicates the number of individual experiments with primary cells of different biological origin. All datasets were normalized to a control group (fold change) and tested for variance. Unpaired Student’s t-test and one-way ANOVA were performed. The probability value is presented in the graphs by a star and follows the probability of error. Where indicated, 95% confidence intervals are reported.

## Results

### Establishment of synthetically designed circRNAs to inhibit miRNAs

**Fig. 1:**
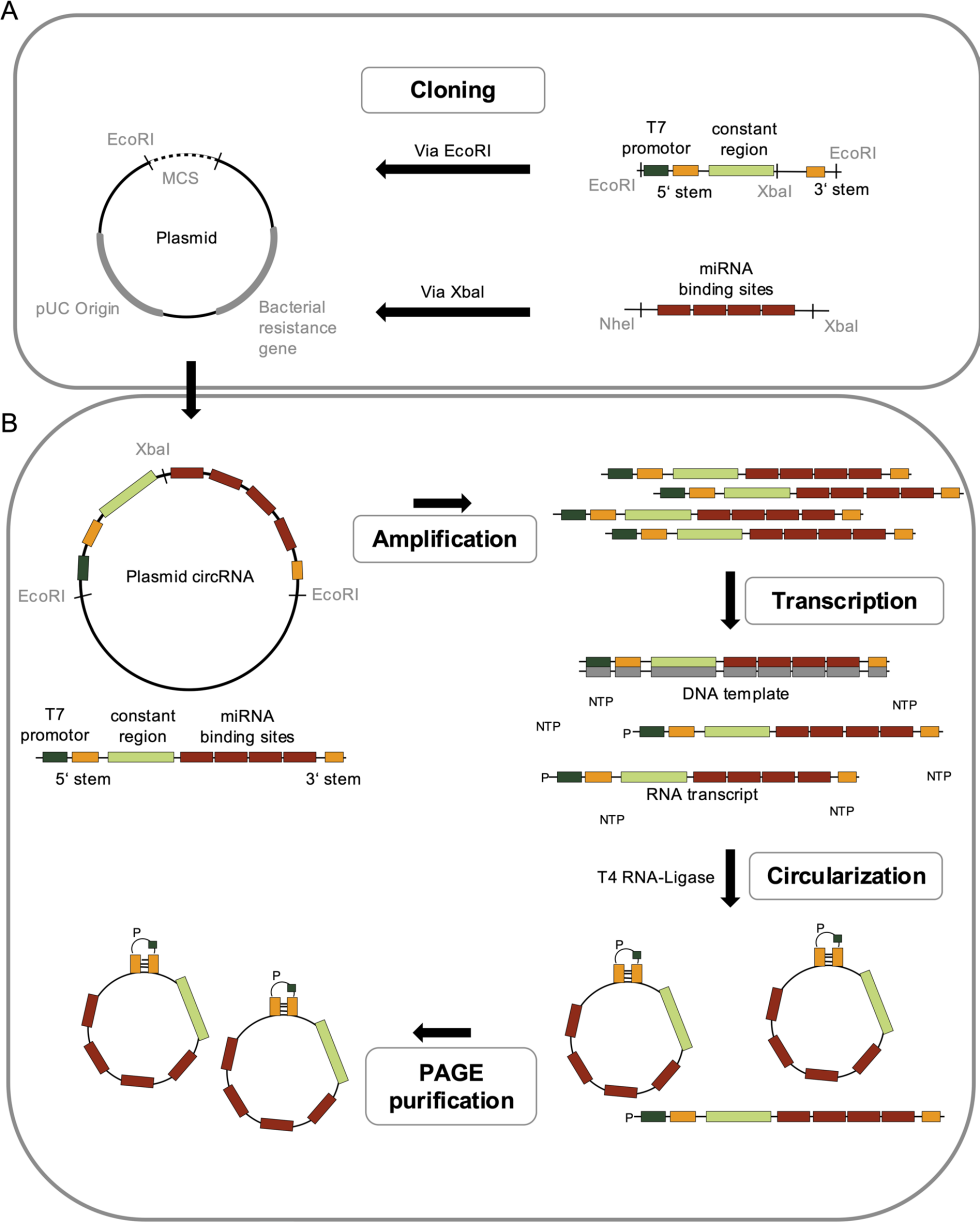
In vitro production of circRNA. The process begins with the cloning procedure (**A**) for circRNA production through insertion of a T7 promoter, double-stranded stem-loop regions, and a constant region via EcoRI, as well as four binding sites for the respective miRNA via XabI. The final circRNA vector (**B**) was amplified, followed by transcription of the DNA template into RNA and DNase digestion to remove template DNA (not shown). Circularization of the RNA is achieved using a T4 RNA-ligase, resulting in both linear and circular RNA products. Therefore, PAGE purification is used to achieve the purest circRNA concentration possible. When both natural degradation processes are compared, circRNA is degraded in a two-step degradation process and cannot be degraded by exonuclease activity due to its circular structure compared to linear anti-miRs.

Synthetic circRNAs were produced through an in vitro protocol involving plasmid-based template generation, transcription, enzymatic circularization, and purification. The cloning process, as can be seen in Fig. 1A, was performed at the Charles Tanford Protein Centre, Halle (Saale), Germany. The cloning strategy used for these constructs involved the insertion of the T7 promoter and constant region into a multipurpose vector backbone via EcoRI restriction sites, followed by the ligation of the designed miRNA binding site array into the vector using XbaI. These cloned plasmids contain all essential sequence elements required for in vitro circRNA synthesis, including: a T7 RNA polymerase promoter for high-yield transcription, a constant region facilitating uniform detection across constructs, and four tandem miRNA binding sites separated by 4 nt spacers. Each binding site was designed as a “bulged” configuration, incorporating a 4 nt mismatch of the target complementarity to mimic physiological RISC interactions and reduce Ago2-mediated cleavage. As illustrated in Fig. 1B, the production process starts with plasmid linearization and amplification. In vitro transcription was carried out using T7 polymerase. The resulting linear RNA was treated with DNase I to remove the DNA template and further subjected to enzymatic ligation using T4 RNA ligase to promote circularization. RNase R exonuclease treatment or PAGE purification can be used to digest linear transcripts and achieve pure circRNA.

**Fig. 2:**
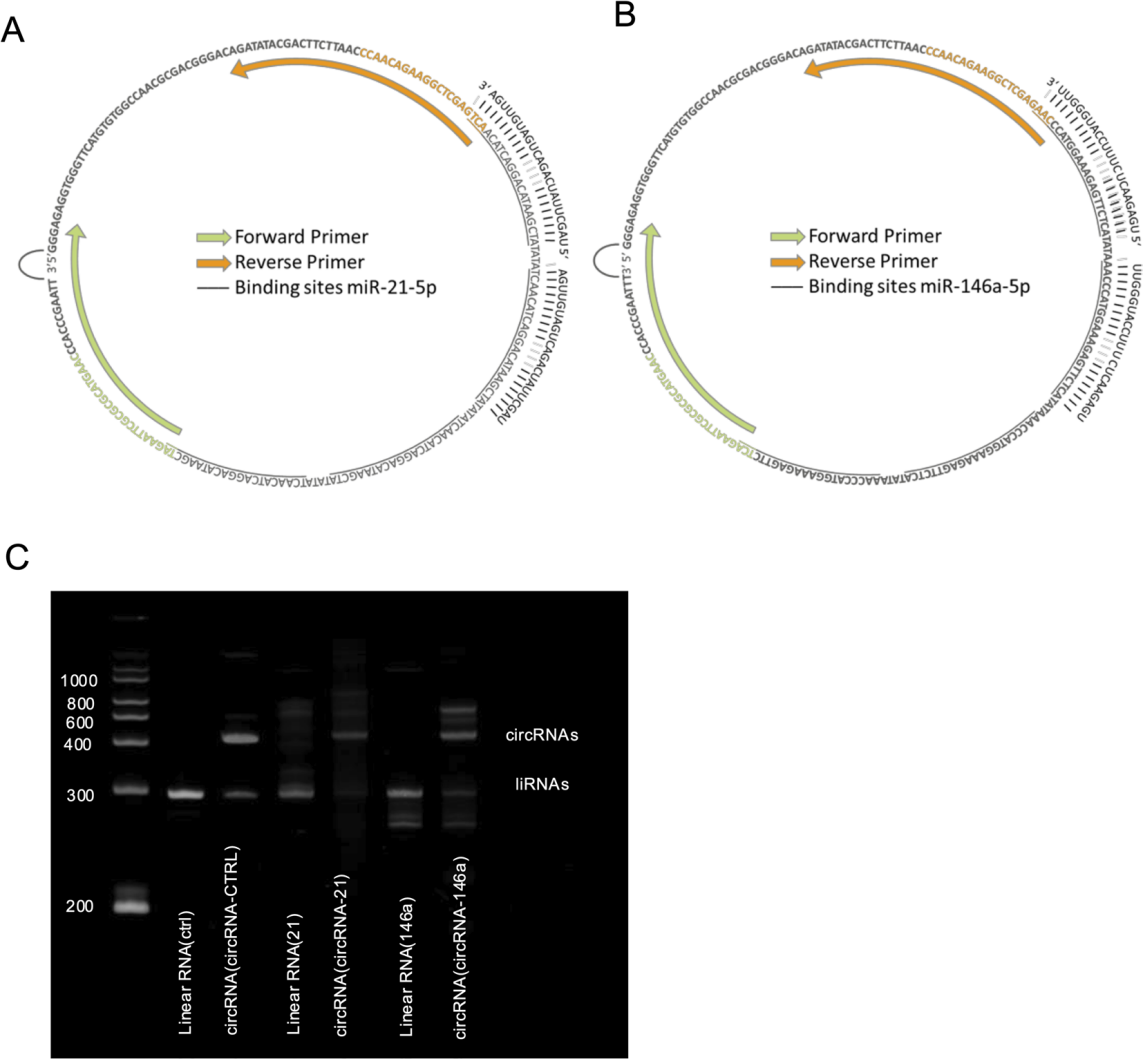
Synthetically designed structure of circRNA-21-5p (**A**) and circRNA-146a-5p (**B**), highlighting the four binding sites for miRNA-21-5p or miRNA-146a-5p (each binding site is underlined) and the primer binding sites for the forward (green) and reverse (orange) Primers. (**C**) Northern blot before PAGE purification with the linear product before the circularization step and post-circularization with the circRNA as well as non-circularized products.

The annotated sequences of circRNA-21 and circRNA-146a are shown in Fig. 2A and B, each containing four binding sites specifically designed to target their respective miRNAs. These binding sites were not perfectly complementary but intentionally constructed as bulged sites by introducing three non-complementary nucleotides. This design mimics the natural binding configuration of miRNAs within the RNA-induced silencing complex (RISC) and has been shown to reduce endonucleolytic cleavage by Argonaute proteins. Primer-binding regions used for RT-PCR validation are indicated.

Northern blot analysis (Fig. 2C) depicts the linear RNA before circularization as well as the RNA products after circularization. Notably, even after RNase R treatment, the presence of linear RNA next to circRNA can be seen. Therefore, polyacrylamide-urea gel purification (PAGE) was used to obtain pure circular RNAs.

**Fig. 3:**
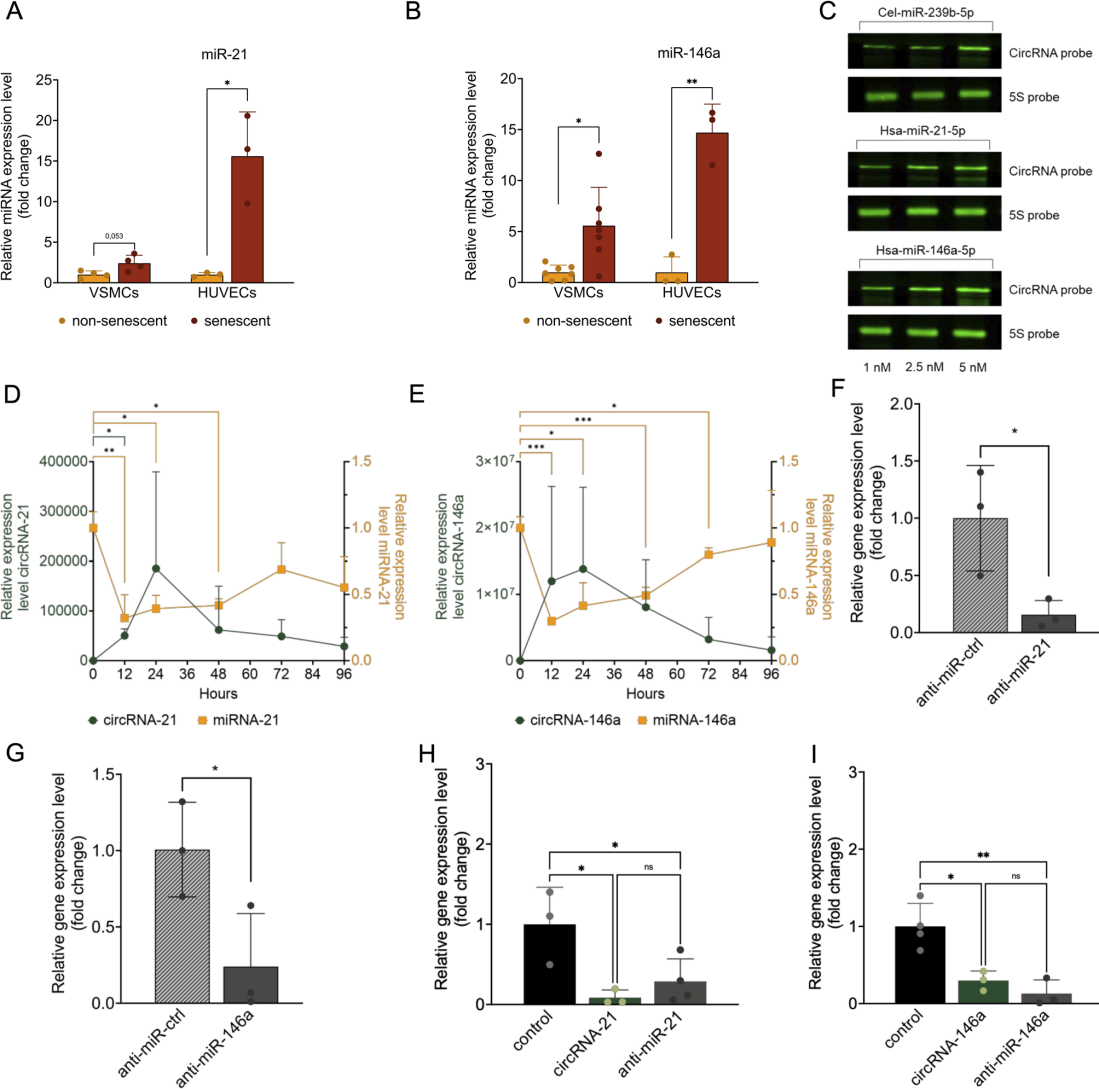
Expression level of miR-21 (**A**) and miR-146a (**B**) is significantly increased in an in vitro model of cellular senescence as detected by miRNA expression analysis, VSMCs and HUVECs, *n* = 3 * *p* < 0.05, ** *p* < 0.01 (CI: VSMC miR-21 ‘-0.1540 to 3.068’ miR-146a ‘1.116 to 8.054’; HUVEC miR-21 ‘1.109 to 28.09’ miR-146a ‘7.959 to 19.46’) (**C**) Northern blot analysis of VSMCs transfected with 1 nM, 2.5 nM, or 5 nM circRNA to determine efficient transfection and circularization of synthetically designed circRNAs. The 5 S Probe was used as a control. Only the relevant band of the Northern blot is shown; the blot was cropped for clarity. The whole image is displayed in Supplementary Fig. 4. (**D**) and (**E**) Time-dependent circRNA and corresponding miRNA concentrations after transfection of either circRNA-21 (**D**) or circRNA-146a (**E**) over 0 to 96 h, *n* = 3, * *p* < 0.05, ** *p* < 0.01, *** *p* < 0.001 (**F**) and (**G**) efficiency of anti-miR transfection, miRNA-21 (**F**), and miRNA-146a expression levels after transfection of either anti-miR-21 (* *p* < 0.05, CI -1.609 to -0.07891) or anti-miR-146a (* *p* < 0.05, CI -1.513 to -0.01989) and anti-miR-ctrl, *n* = 3. Data are presented as mean ± SD relative to control. (**H**) and (**I**) depict miR-146a and miR-21 levels after transfection with either 2.5 nM circRNA or 50 nM anti-miR in VSMC. MiRNA levels are significantly reduced upon transfection to a comparable level despite lower concentrations of circRNA used, *n* = 3–4, * *p* < 0.05, ** *p* < 0.01.

To investigate the cellular effects of circRNA-mediated miRNA inhibition under physiologically relevant conditions, experiments were conducted in a cellular context in which the target miRNAs are endogenously upregulated. Early replicative senescence was selected as the model system, as it reflects key features of vascular aging, where miR-21-5p and miR-146a-5p are described to be elevated. This approach allows for the assessment of inhibitory effects under conditions that approximate pathophysiological relevance, without placing the primary focus of the study on senescence itself. To verify our model system, quantitative RT-PCR was used to analyze miRNA expression. Expression of miR-21-5p and miR-146a-5p was increased in late-passage compared to early-passage cells across all vascular cell types (Fig. 3A, B), with statistical significance observed (*p*≤0.05, respectively, *p* < 0.01).

In cells transfected with increasing concentrations of circRNA (1 nM, 2.5 nM, and 5 nM), a dose-dependent detection of circRNA was observed (Fig. 3C). 5 S rRNA served as a normalization control.

To assess the temporal dynamics of circRNA expression and its effect on target miRNA levels, VSMCs were transfected with either circRNA-21 or circRNA-146a and analyzed over 96 h. As shown in Fig. 3D, E, expression levels of both circRNAs peaked at around 24 h post-transfection and declined progressively over time. In parallel, miRNA expression was measured to evaluate its availability in response to circRNA transfection. For both miR-21-5p and miR-146a-5p, relative expression gradually decreased over time, especially in the first 24 h, with a significant reduction in miRNA levels after circRNA transfection. Both miRNAs did reach baseline levels after 96 h. These measurements provide a time-resolved profile of circRNA uptake and miRNA modulation in VSMCs. Further, transfection efficiency and miRNA-modulation were assessed by northern blotting (Supplemental Fig. 2). To compare the effect of circRNA transfection to the impact of anti-miR transfection, inhibition efficiency was tested for anti-miR-21 and anti-miR-146a in VSMCs. Compared to the anti-miR-control (anti-miR-ctrl), anti-miR-21 as well as anti-miR-146a show a significant reduction of miRNA expression 48 h after transfection of 50 nM of the respective anti-miR. Further, miR-146a and miR-21 levels were measured after transfection with either 2.5 nM circRNA or 50 nM anti-miR in VSMC. MiRNA levels are significantly reduced upon transfection to a comparable level despite lower concentrations of circRNA used (Fig. 3H, I). To further study miRNA and circRNA interaction, cells were transfected with precursor-miRNA in the presence of circRNA. Co-transfection with circRNA-146a attenuated the pre-miR-146a–induced change in mature miR-146a levels, consistent with a miRNA-dependent functional interaction (Supplemental Fig. 5).

Taken together, these data demonstrate the successful production, detection, and cellular delivery of synthetic circRNAs in vascular cells under controlled experimental conditions.

### CircRNAs influence miRNA-21 target regulation in VSMCs

**Fig. 4:**
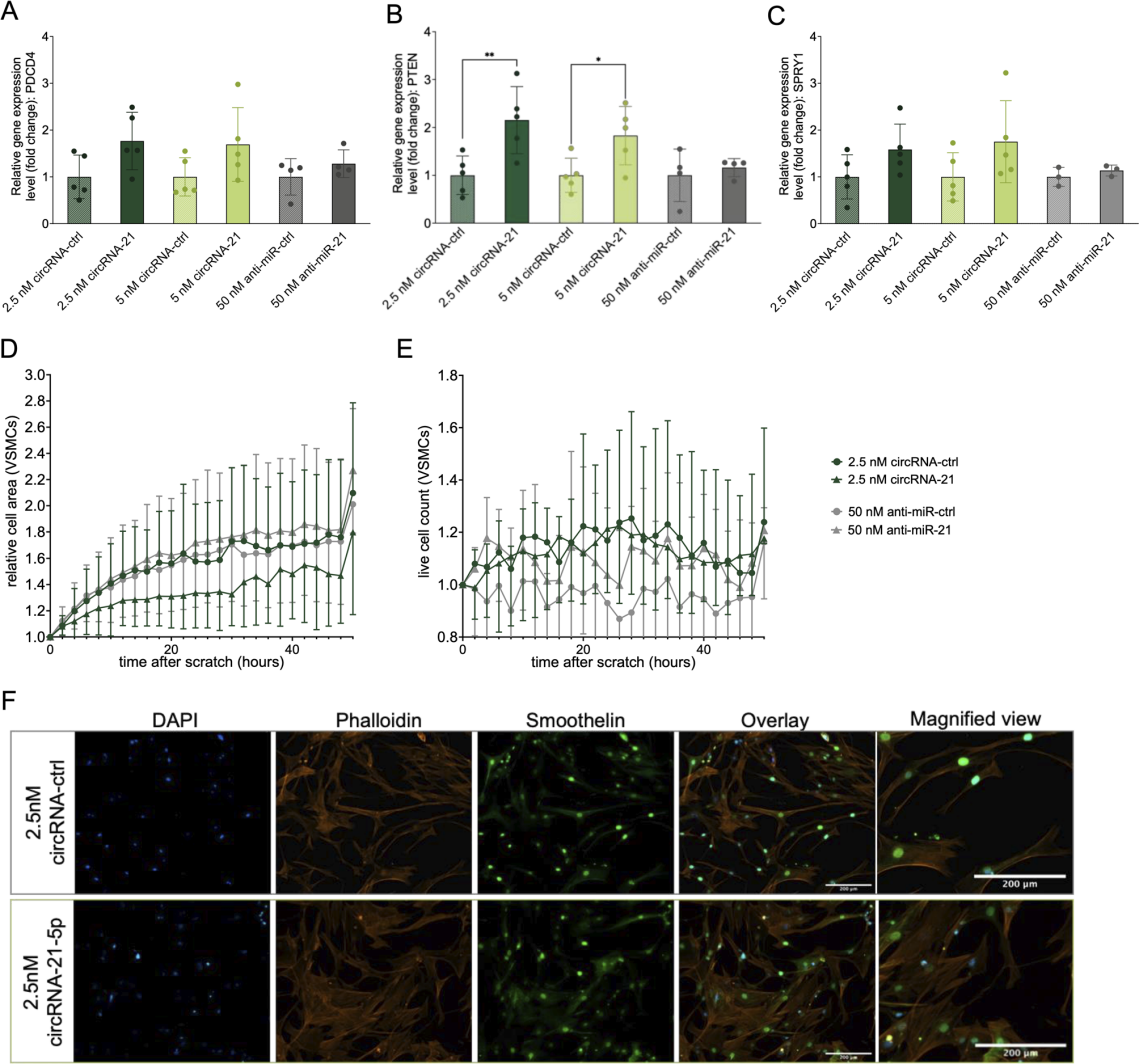
CircRNAs influence miR-21-5p target regulation and cellular function of VSMCs. (**A**)–(**C**) (qRT)PCR analysis of miR-21-5p targets PDCD4 (left), PTEN (middle), and SPRY1 (right). Cells were transfected with either 2.5 nM, 5 nM circRNA, or 50 nM anti-miR in senescent VSMCs, *n* = 5, * *p* < 0.05. (CI for PDCD4: 2.5nM ‘– 1.637 to 0.1050’, 5nM ‘– 1.564 to 0.1778’ and 50nM anti-miR ‘– 1.255 to 0.6928’; PTEN: 2.5 nM ‘– 1.979 to – 0.3287’, 5nM ‘– 1.653 to – 0.003237’ and 50nM anti-miR ‘– 1.083 to 0.7621’; SPRY1 2.5 nM ‘– 0.2748 to 1.443’, 5nM ‘– 0.1072 to 1.611’ and 50nM anti-miR ‘– 1.331 to 1.059’) (**C**) The effect of circRNAs on cellular function is measured by migration capacity. Migration capacity was determined via scratch wound assay in VSMCs to inhibit miR-21-5p through circRNAs, *n* = 4. (**E**) The effect of circRNAs on cellular function measured by proliferation. Proliferation was determined by cell counting in life cell imaging of senescent VSMCs to inhibit miR-21-5p through circRNAs, *n* = 4. (**F**) Immunofluorescence staining of replicative senescent cells, VSMCs transfected with either 2.5 nM circRNA-21-5p or 2.5 nM circRNA-control with staining for DAPI (blue), Phalloidin (red), and Smoothelin (green). Images were acquired at 10× magnification. Scale bar 200 μm. Data are presented as mean ± SD relative to control.

To assess the effect of circRNA-21 on miRNA-21-5p target gene expression, cells were transfected with either 2.5 nM or 5 nM of circRNA-21 or, in comparison, with 50 nM anti-miR-21. Expression of three previously validated direct miR-21-5p targets - PDCD4, PTEN, and SPRY1 - was analyzed by qRT-PCR 48 h post-transfection (Fig. 4A–C). The most pronounced effect was seen for PTEN, which exhibited a statistically significant increase in expression at 5 nM circRNA-21. PDCD4 and SPRY1 also showed a consistent upward trend, though without statistical significance at the tested doses. In contrast, cells treated with 50 nM anti-miR-21 did not show a comparable level of target gene regulation. Across all three targets, mRNA levels following anti-miR treatment remained lower than those observed after 5 nM circRNA transfection, indicating a differential effect of the two inhibition strategies under the tested conditions. Protein-level validation by western blot confirmed regulation of downstream targets following circRNA- and anti-miR–mediated miRNA inhibition (Supplementary Fig. 6).

In addition to target regulation, cellular function was assessed. Cells treated with circRNA-21 exhibited a decrease in migration behavior compared to control-transfected cells (Fig. 4D, E, Supplementary Fig. 7). In contrast, VSMCs transfected with anti-miR-21 do not show a difference in migration capacity compared to the control group.

Figure 4D, E present proliferation data derived from live-cell imaging, showing relative changes in cell count over 48 h. Cells transfected with circRNA-21 do not show a difference in proliferation compared to the control, whereas cells transfected with anti-miR-21 show a slight reduction in proliferation capacity.

To visualize structural features of transfected cells, immunofluorescence staining was performed 48 h after transfection with 2.5 nM circRNA-21 or circRNA-control. As shown in Fig. 4F, staining for DAPI, Phalloidin, and Smoothelin revealed no difference in cytoskeletal organization and contractile protein expression among treated groups. The same experiments were also conducted in endothelial cells and can be seen in Supplementary Fig. 8.

### CircRNAs influence miRNA-146a target regulation in VSMCs

**Fig. 5:**
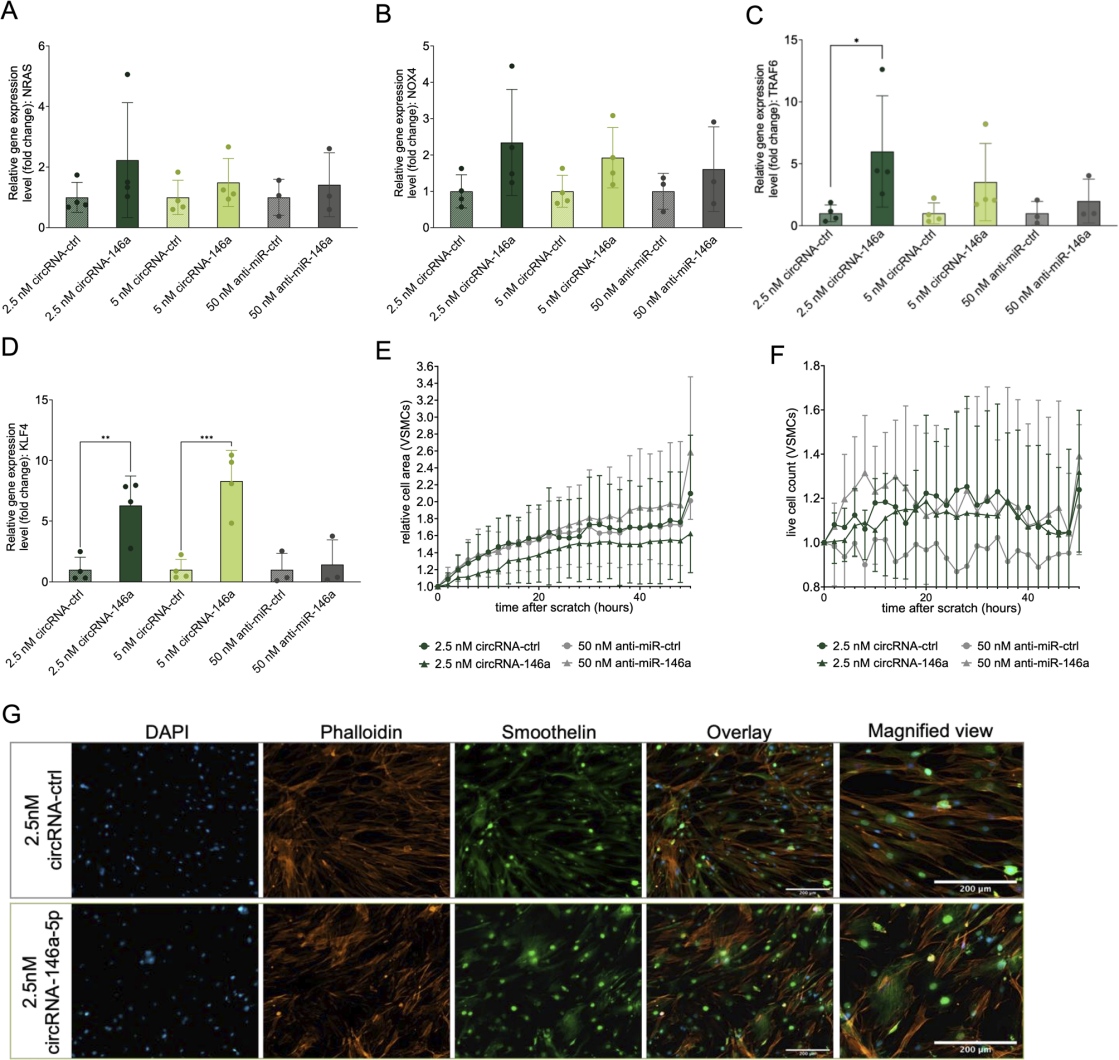
CircRNAs influence miR-146a-5p target regulation and cellular function of VSMCs. (**A**)–(**D**) (qRT)PCR analysis of miR-146a-5p targets NRAS, NOX4, TRAF6, KLF4. Cells were transfected with either 2.5 nM, 5 nM circRNA, or 50 nM anti-miR in senescent VSMCs, *n* = 4, *** *p* < 0.001, **** *p* < 0.0001. (CI for NRAS: 2.5nM ‘– 0.7300 to 3.189’, 5nM ‘– 1.468 to 2.451’ and 50nM anti-miR ‘– 1.846 to 2.679’; NOX4: 2.5 nM ‘– 0.3464 to 3.032’, 5nM ‘– 0.7644 to 2.614’ and 50nM anti-miR ‘– 1.341 to 2.561’; TRAF6 2.5 nM ‘0.2419 to 9.737’, 5nM ‘– 2.226 to 7.269’ and 50nM anti-miR ‘-4.496 to 6.468’; KLF4 2.5 nM ‘1.820 to 8.748’, 5nM ‘3.839 to 10.77’ and 50nM anti-miR ‘– 3.566 to 4.433’) (**E**) The effect of circRNAs on cellular function is measured by migration capacity. Migration capacity was determined via scratch wound assay in VSMCs to inhibit miR-146a-5p through circRNAs, *n* = 4. (**F**) The effect of circRNAs on cellular function measured by proliferation. Proliferation was determined by cell counting in life cell imaging of senescent VSMCs to inhibit miR-146a-5p through circRNAs *n* = 4. (**G**) Immunofluorescence staining of replicative senescent cells VSMCs transfected with either 2.5 nM circRNA-146a-5p or 2.5 nM circRNA-control, with staining for DAPI (blue), Phalloidin (red), and Smoothelin (green). Images were acquired at 10× magnification. Scale bar 200 μm. Data are presented as mean ± SD relative to control.

To evaluate the regulatory effects of circRNA-146a, the same experiments as for circRNA-21 were designed with 50 nM anti-miR-146a as a control group. Quantitative RT-PCR was performed 48 h post-transfection to assess mRNA levels of six established targets: NRAS, NOX4, TRAF6, and KLF4. As shown in Fig. 5A–D, all four genes exhibited an increase in expression following circRNA-146a treatment, predominantly after the transfection of 2.5 nM circRNA-146a. For KLF4, the upregulation reached statistical significance at both concentrations, with the most pronounced effects observed at 5 nM. In contrast, transfection with 50 nM anti-miR-146a resulted in either a weaker or no comparable increase in target expression.

Protein-level validation by western blot confirmed regulation of downstream targets following circRNA- and anti-miR–mediated miRNA inhibition (Supplementary Fig. 6).

Migration capacity in circRNA-146a-treated VSMCs (Fig. 5E) did not differ notably from control-transfected cells, showing a slight downregulation after circRNA transfection, whereas anti-miR-146a transfection showed a slight upregulation compared to the control group. For proliferation (Fig. 5F), no consistent differences were detected between circRNA-146a and the respective control. However, anti-miR-146a-treated VSMCs showed an increase in proliferation compared to the respective controls. Further, no morphological alterations or differences in contractile protein expression were observed between the groups (Fig. 5G).

Again, the same set of experiments was also performed in endothelial cells, as shown in Supplementary Fig. 9. Here, especially the functional assays showed changes following circRNA-146a transfection.

## Discussion

Endogenous circRNAs are abundantly expressed in eukaryotic cells and have been shown to fulfill diverse regulatory roles. One of their most well-characterized functions is the sequestration of miRNAs through complementary binding, thereby functioning as molecular sponges. A well-characterized example is the neuronal circRNA CDR1, which contains more than 60 conserved binding sites for miR-7, effectively modulating miR-7 activity and its downstream gene targets^25,26^. The sponge-like behavior of circRNAs with the sequestration of miRNAs offers a natural blueprint for the design of synthetic circRNAs capable of targeted miRNA inhibition.

This study aimed to investigate the inhibition of miR-21-5p and miR-146a-5p using synthetically engineered circular RNAs (circRNAs) in human vascular cells. These miRNAs are known to be upregulated in vascular aging and contribute to pathophysiological processes such as inflammation, fibrosis, and remodeling, making them clinically relevant targets for therapeutic modulation^5,6^. It is also the first study to investigate whether these synthetically designed circRNAs are suitable for reaching and functionally influencing hard-to-transfect vascular cells.

To this end, we designed synthetic circRNAs containing four bulged miRNA binding sites, a configuration that mimics physiological miRNA-mRNA interactions within the RNA-induced silencing complex (RISC) and prevents Argonaute 2-mediated cleavage. This approach has been shown to enhance sponge stability and prolong inhibitory activity, as previously demonstrated for miR-122 and miR-21 sponges^18,19^.

CircRNAs were synthesized through an optimized in vitro process involving T7 polymerase-driven transcription, enzymatic ligation using T4 RNA ligase, and PAGE-based purification to concentrate the circularized product. This strategy is consistent with current best practices in circRNA production technologies and takes advantage of GMP-initiated transcription to avoid dephosphorylation steps^18,27^. Among available circularization strategies, enzymatic ligation remains one of the most efficient and scalable methods for generating non-coding circRNAs of small to moderate length, including miRNA sponges^28^. Challenges remain in achieving pharmaceutical-grade purity, ensuring batch-to-batch consistency, and achieving cost-effective large-scale production^29,30^. Lyophilization strategies have shown promise for extending shelf life and eliminating the need for ultra-low-temperature storage^31^.

Performance of northern blot analysis, as done here with the final products as well as with transfected cells, is widely accepted to study and confirm the circularization process, as circularized products display a different separation within the electrophoresis gel^32^.

The kinetics of circRNA and miRNA levels were evaluated over 96 h post-transfection, where circRNA expression peaked within the first 24 h and gradually declined over time, while target miRNA levels displayed an inverse pattern, reaching a minimum at early time points and returning toward baseline at later stages. This time-dependent relationship is in agreement with previous studies showing that synthetic circRNAs can induce robust, but temporally limited, miRNA repression^27,28^. These dynamics likely reflect both the gradual degradation of circRNA molecules and the shifting intracellular balance between free and bound miRNA.

Our findings confirm the effective design, synthesis, and delivery of synthetic circRNAs targeting miR-21-5p and miR-146a-5p in a disease-relevant vascular context. Further, co-transfection of vascular cells with pre-miR-146a and circRNA-146a shows a significant reduction of miRNA-146a levels compared to the transfection of pre-miR-146a and circRNA-control (Supplementary Fig. 5). These results demonstrate that circRNA-146a can functionally counteract the activity of excess miR-146a, providing independent evidence that circRNA-mediated effects are miRNA-dependent and occur upstream of the target gene regulation. While this data does not constitute direct biochemical proof of physical binding, they provide strong functional evidence consistent with a direct circRNA–miRNA interaction.

Compared to linear antisense oligonucleotides (anti-miR), circRNAs offer greater structural stability, tunability, and intracellular persistence, making them a promising platform for future RNA-based therapeutics in cardiovascular disease^18,32^.

For circRNA-based miRNA sponges, off-target effects are a significant concern that requires careful validation. While circRNAs can effectively sequester target miRNAs, several issues arise: (1) most circRNAs lack the multiple high-affinity binding sites necessary for efficient miRNA sponging, and (2) miRNA sponging can affect multiple downstream targets beyond intended pathways. The circRNAs used in this study were engineered to contain four tandem bulged binding sites specific for either miR-21-5p or miR-146a-5p. The number of binding sites was deliberately selected to balance sequestration efficiency with structural stability. Importantly, bulged binding sites were used rather than perfectly complementary sequences. This design mimics endogenous miRNA-mRNA interactions and prevents AGO2-mediated endonucleolytic cleavage, thereby stabilizing the circRNA while avoiding irreversible miRNA degradation^33,34^. To ensure the specificity of the circRNAs, the BLAST database was used to predict potential off-target interactions^35^.

Another important consideration for translational application is immunogenicity. Unmodified exogenous circRNA can bypass cellular RNA sensors (RIG-I and TLRs) and avoid provoking immune responses when highly purified, showing reduced immunogenicity compared to linear mRNA^36^. However, purity is critical: even small amounts of contaminating linear RNA can trigger robust immune responses^36^. Therefore, we performed additional purification using PAGE to minimize linear RNA contamination.

The efficiency of anti-miR-21 and anti-miR146a was assessed to ensure that transfection was successful and to confirm sufficient miRNA inhibition. Both miRNA-21 and miRNA-146a expression levels were significantly reduced after anti-miR transfection. This is relevant to be able to compare the effect of circRNA versus anti-miR. The observation that circRNA-21 and anti-miR-21 reduce mature miR-21 levels to a similar extentsuggests that the distinct cellular responses observed are not driven by quantitative differences in miRNA depletion. Instead, these findings point toward qualitative differences in how distinct inhibitory strategies interfere with miRNA function. A systematic comparison of potency and intracellular kinetics will require dedicated future studies.

To further evaluate the biological relevance of synthetic circRNA-mediated miRNA inhibition, we analyzed the expression of target genes and key cellular behaviors in vascular cells. CircRNA-21 and circRNA-146a were transfected at physiologically relevant concentrations, and their effects were compared to anti-miRs.

For miR-21-5p inhibition, expression of its validated targets PTEN, PDCD4, and SPRY1 was assessed, showing increased mRNA levels following circRNA-21 transfection. The observed regulation is in line with previous findings reporting the upregulation of PTEN, PDCD4, and SPRY1 after inhibition of miR-21-5p^10–12,37^. This was further supported on the protein level using Western Blot analysis (Supplementary Fig. 6). Notably, the anti-miR treatment did not elicit comparable changes, despite being used at one-tenth the concentration. Similar results were observed in target regulation after the transfection of circRNA-146a. These results support previous findings that bulged circRNA sponges more effectively inhibit miRNA activity than linear antisense inhibitors, likely due to enhanced intracellular stability and sustained sequestration^18^.

To assess whether circRNA-mediated miRNA inhibition impacts key cellular behaviors, we analyzed migration, proliferation, and cytoskeletal integrity in VSMCs following transfection. These functions are particularly relevant given the established roles of miR-21-5p and miR-146a-5p in promoting VSMC activation, motility, and phenotypic switching in vascular remodeling^38–40^.

Migration assays revealed that inhibition of miR-21-5p by circRNA-21 mildly reduced VSMC motility, a finding consistent with the known anti-migratory role of PTEN and SPRY1^12,38^. In contrast, anti-miR-treated cells showed no change in migration, indicating a potentially stronger or more sustained inhibitory effect of circRNAs at lower doses.

For miR-146a, a mild reduction in migration was observed after circRNA-146a transfection. However, the effect was less pronounced than with miR-21 targeting, possibly due to the more nuanced regulatory role of miR-146a in cytoskeletal dynamics. In proliferation, circRNA-treated VSMCs exhibited largely unchanged growth kinetics, while anti-miR-21 showed a slight reduction in proliferation. A discrepancy that may reflect differences in the uptake or intracellular persistence of the RNA species. Although miR-21 and miR-146a have been linked to cell cycle progression through repression of tumor suppressors and transcription factors such as PDCD4, PTEN, and KLF4^10,11,15,41^, the observed target regulation may not have been sufficient to override proliferation-maintaining signals in the VSMCs, especially in their early-senescent cell state. To further determine whether circRNA altered the phenotypic state or structural characteristics of VSMCs, we performed immunofluorescence staining, where no differences in cytoskeletal organization or contractile protein expression were observable.

Together, these findings demonstrate that synthetic circRNAs not only recapitulate the miRNA sponge function observed in endogenous circRNAs such as CDR1^21^ but also outperform conventional antisense strategies in primary vascular cells. Their impact on key regulatory targets and cell behavior highlights their therapeutic promise for modulating vascular remodeling and inflammation in disease contexts where miR-21-5p and miR-146a-5p are pathologically upregulated.

In addition to VSMCs, circRNA-21 and circRNA-146a were tested in endothelial cells to evaluate their regulatory capacity, where both miRNAs are also known to be upregulated with age^4,6,15,42^. In contrast to VSMCs, only modest changes in target gene expression were observed, whereas cellular functions were shown to be more affected compared to VSMCs. Endothelial cell behavior, particularly migration and proliferation, could be modulated by subtle post-transcriptional changes, protein-level regulation, or shifts in cytoskeletal organization that are not fully captured at the mRNA level. Additionally, miR-21 and miR-146a have been implicated in endothelial repair and angiogenesis via multiple pathways, including PI3K/AKT^37^ signaling, where small changes in regulatory node activity may be sufficient to improve functional performance. The improved migration and proliferation observed here may reflect a rapid, post-transcriptional rebalancing of signaling networks rather than large transcriptional changes in canonical miRNA targets.

Differences in transfection efficiency or RNA uptake between ECs and VSMCs could contribute to lower intracellular circRNA concentrations in endothelial cells, limiting the extent of miRNA sequestration. Moreover, the baseline expression of miRNA targets may differ between cell types, and some targets in ECs may already be expressed at minimal levels, reducing the observable impact of circRNA-mediated target regulation. These factors highlight the cell-type specificity of miRNA function and emphasize the need to tailor circRNA designs to the biological context of interest.

Even though this study demonstrates the feasibility and functional relevance of using synthetic circular RNAs to inhibit disease-associated microRNAs in vascular cells, we also want to acknowledge their limitations. By targeting miR-21-5p and miR-146a-5p, we show that circRNAs can modulate target gene expression and specific cellular functions, especially in VSMCs. However, due to the low sample sizes and high standard deviations when working with primary cells, the level of classic statistical significance was not always reached. The use of small sample numbers of biological replicates is common in mechanistic studies using primary vascular cells. While consistent effects were observed across independent experiments, larger sample sizes will be required in future studies to further validate these findings. Further, to be able to visualize possible effects of miRNA inhibition, we decided to work with a model in which these are already physiologically or age-relatedly upregulated. Early replicative senescence was selected as the model for this study because it recapitulates the key molecular and phenotypic features of in vivo vascular aging, including activation of the p53/p21 and p16/Rb pathways, acquisition of the senescence-associated secretory phenotype (SASP), and morphological changes characteristic of senescent vascular cells^43,44^. Early senescent cells retain sufficient viability and responsiveness to therapeutic interventions, providing a clinically relevant window for assessing whether miRNA inhibition can reverse or attenuate the senescent phenotype^45,46^. Senescence in our model was not defined solely by passage number. Instead, senescent status was validated using established molecular markers, including p16INK4A, p21, p14ARF, and LAMIN B1 (Supplementary Fig. 1), in line with commonly accepted criteria for characterizing vascular cell senescence^45^.

However, this choice of model system may have influenced and also overshadowed other positive and negative effects of circRNA inhibition. Future studies should therefore definitely consider these circumstances. To counteract this limitation, it was therefore also important for us to investigate the effect of more than one synthetic circRNA and in more than one cell type to be able to draw general conclusions.

Furthermore, efficient delivery remains a central challenge for the clinical translation of circRNA therapeutics. Lipid nanoparticles (LNPs) represent the most advanced delivery platform for circRNA therapeutics, with recent studies demonstrating successful in vivo delivery and sustained protein expression^47,48^. For cardiovascular applications, various non-viral delivery systems, including extracellular vesicles, polymeric nanoparticles, and hydrogels, have been explored for RNA therapeutics in VSMCs and endothelial cells^49^. However, delivery efficiency to cardiovascular tissues remains a major challenge, particularly in achieving adequate cellular uptake in vascular cells while minimizing off-target accumulation^48^.

The present study was designed as a mechanistic in vitro proof-of-concept to assess circRNA-mediated miRNA inhibition in senescent human vascular cells. While consistent effects were observed across two independently designed circRNAs and two primary cell types, in vivo validation will be required in future studies to evaluate delivery, biodistribution, and therapeutic potential.

Taken together, compared to conventional anti-miRs, circRNAs offer advantages in stability, dosing efficiency, and sustained inhibitory capacity. Importantly, their effects are cell-type specific, emphasizing the need for context-aware design in RNA-based therapies. As synthetic circRNAs are further refined, including potential improvements in delivery systems and circularization strategies, they may represent a novel and versatile platform for therapeutic intervention in cardiovascular and other age-related diseases.

## Supplementary Information

Below is the link to the electronic supplementary material.

Supplementary Material 1

## Data Availability

The datasets used and analyzed during the current study are available from the corresponding author on reasonable request.
