# Supplementary material for: Synthetically designed circRNA can be used to target cardiovascular relevant microRNAs to improve cellular function
Source: Sci Rep. 2026 Apr 9;16:12023. doi: 10.1038/s41598-026-46870-7 (PMC13068899; doi:10.1038/s41598-026-46870-7)
Supplement: Supplementary file 1 — Supplementary Material 1 [file 41598_2026_46870_MOESM1_ESM.pdf]

Supplementary Material and Methods

Supplementary Table 1- Overview of primers used

All primers listed below were purchased from Eurofins Genomics (Luxemburg, Luxemburg)

Primer	Sequence (forward)	Sequence (reverse)
CircRNA-146a	TCAGAATTCGCGCGCATGAAC	GTTCTCGAGCCTTCTGTTGG
CircRNA-21	TAGAATTCGCGCGCATGAAC	TGACTCGAGCCTTCTGTTGG
CircRNA-Ctrl	CCAACAGAAGGCTCGAGCAG	TTCATGCGCGCGAATTCTTT
FBXO7	GCTCGCACCTGAGGCAGTCC	GTCTCTTCATCTCCAGTGAGGGG
GAPDH	TGCACCACCAACTGCTTAGC	GGCATGGACTGTGGTCATGAG
IRAK1	CACACTGCAAGCAGGTCTGGCT	GGACACGTAGGAGTTCTCCTGGGTC
KLF4	TCTCAAGGCACACCTGCGAA	TAGTGCCTGGTCAGTTCATC
NOTCH1	CAGAGCGGCATGGTGCCGAA	GCGCTGGCAGCAAGGCTACT
NOX4	TCCACCAGATGTTGGGGCTAGGA	AGCCAAGAGTGTTCGGCACATGG
NRAS	TTCATGGCGGTTCCGGGGTC	ACCAGCAAGAACCTCAAGCTCCA
pcircRNAi	TAATACGACTCACTATAGGGAGAGGTGGG (sense)	AATTCGGGTGGGTTCATGCG (anti-sense)
PDCD4	GCAAAAAGGCGACTAAGGAAAAA	TAAGGGCGTCACTCCCACT
PTEN	TGTGGTCTGCCAGCTAAAGG	CGGCTGAGGGAACTCAAAGT
SPRY1	GCAGTGGCAGTTCGTTAGTTG	CAGTAGGCTGAATCTCTCTCTCA
TRAF6	CCCGCGCACTAGAACGATCATG	GCTGGATCCACAGCTGTTTTCACA

Supplementary Table 2 - List of used antibodies for immunofluorescence microscopy

Antibody	Reference	Manufacturer	Dilution
Phalloidin-TRITC	P1951	Sigma Aldrich, Hamburg, Germany	1:100
Smoothelin	Smoothelin (H-300) sc-28562	Santa Cruz Biotechnology, Dallas, USA	1:100
CD31	#3528S	Cell Signaling	1:100

Supplementary Method 1: Western Blot

Protein samples (30 µg per lane) were mixed with loading dye (Roti®-Load, Carl Roth) and separated on 7.5% precast polyacrylamide gels (Bio-Rad). Precision Plus Protein™ Dual Color Standards (Bio-Rad) were used as molecular weight markers. Electrophoresis was performed at 90 V for 100 min using a Bio-Rad tank blot system.
Proteins were transferred onto PVDF membranes using transfer buffer at 90 V for 1 h. Membranes were blocked in 5% non-fat dry milk dissolved in TBS-T for 1 h at room temperature and subsequently incubated with primary antibodies diluted in TBS-T overnight at 4 °C. After washing three times with TBS-T, membranes were incubated with HRP-conjugated secondary antibodies for 2 h at room temperature. Following additional washing steps, protein bands were visualized using enhanced chemiluminescence (ECL) and detected with a ChemiDoc™ imaging system (Bio-Rad).
(ab72543) Anti-KLF4 antibody, Abcam; PDCD4 (D29C6) XP® Rabbit mAb #9535, Cell Signaling Technology; PTEN (D4.3) XP® Rabbit mAb #9188, Cell Signaling Technology; TRAF6 (D21G3) Rabbit mAb #8028, Cell Signaling Technology; Spry1 (D9V6P) Rabbit mAb #13013, Cell Signaling Technology.

Supplementary Figure 1: Quantification of senescence-associated genes p16INK4A, p21, p14ARF, and Lamin B1 in low and high passage numbers of VSMC to verify the replicative senescence model as described previously. n=3, *p<0.05, **p<0.01

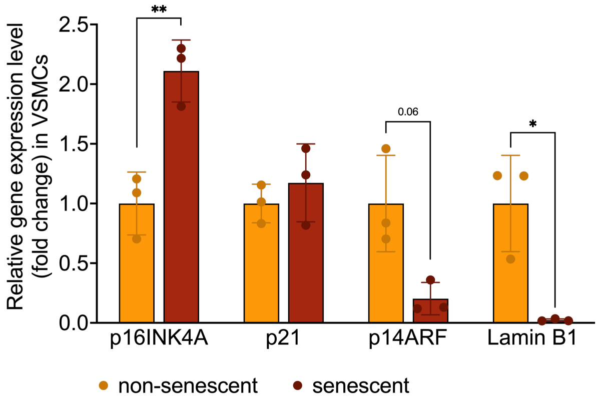

Supplementary Figure 2: pcSponge Design for circRNA Production

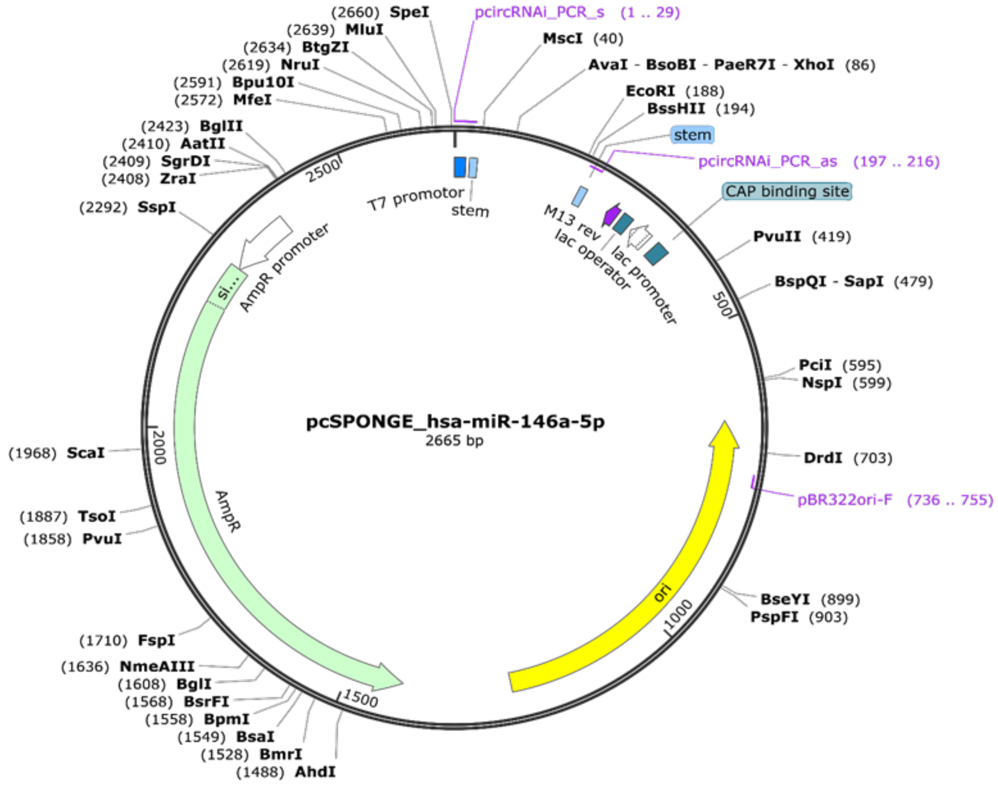

The pcSponge for the circRNA-146a production is shown here. A designed oligo consisting of four binding sites for miR-146a-5p spaced with tata boxes between each binding site. Further, the oligos sense strand starts with a recognition site for Xho1 and the anti-sense strand with a recognition site for EcoRI. This part of the plasmid, starting with the binding site for the pcircRNAi_PCR_sense primer at the T7 promotor region, and reaching the pcircRNAi_PCR_anti-sense primer sequence at the stem sequence, can be replicated using PCR. These PCR products can then be used to circularise and form circRNAs.

Supplementary Figure 3: Northern Blot of HUVECs transfected with either 2.5 nM or 5 nM circRNA-146a or circRNA-control (48 hours). A miRNA-146a probe was used to detect miRNA-146a levels. U6 was used as a housekeeper.

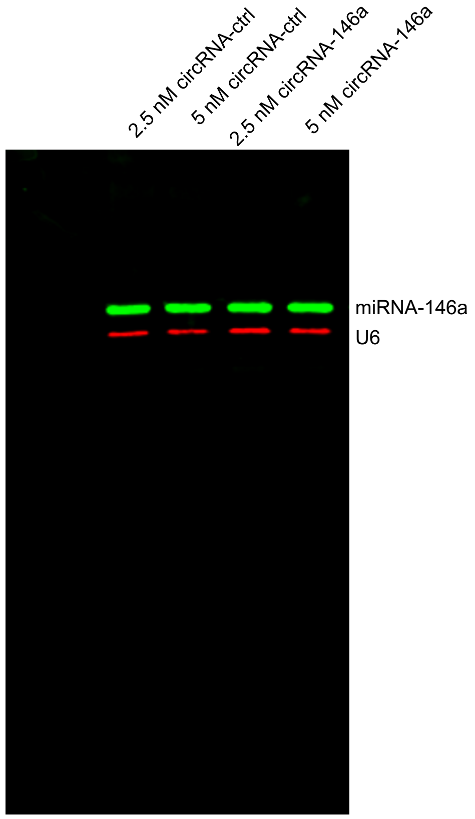

Supplementary Figure 4: Whole image of Northern blot analysis displayed in Figure 3C of VSMCs transfected with 1 nM, 2.5 nM, or 5 nM circRNA to determine efficient transfection and circularization of synthetically designed circRNAs. 5S Probe was used as a control.

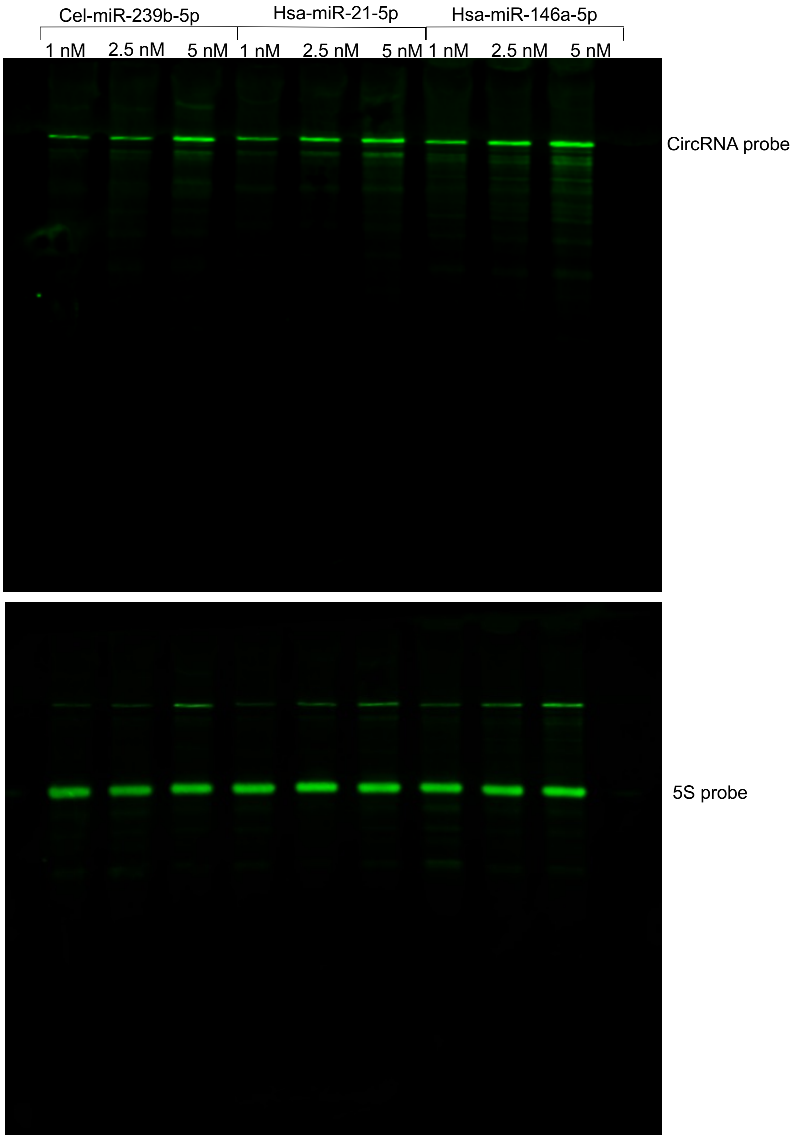

Supplementary Figure 5: Effect of circRNA-146a on pre-miR-146a–mediated regulation of mature miR-146a levels. Transfection of VSMC with circRNAs, and pre-miRs was performed using Lipofectamine RNAiMAX (Thermo Fisher Scientific) in Opti-MEM (Thermo Fisher Scientific) according to the manufacturer's instructions. Final concentrations of circRNAs were 2.5 nM, and pre-miRs (Thermo Fisher Scientific) were transfected at a final concentration of 5 nM, as indicated. A) VSMC and B) HUVEC were transfected with control pre-miR or pre-miR-146a in the presence of circRNA-146a (2.5 nM), as indicated. miR-146a levels were quantified by RT–qPCR. Co-transfection with circRNA-146a attenuated the pre-miR-146a–induced change in mature miR-146a levels, consistent with a miRNA-dependent functional interaction. n=3, *p<0.05, **p<0.01, ***p<0.001

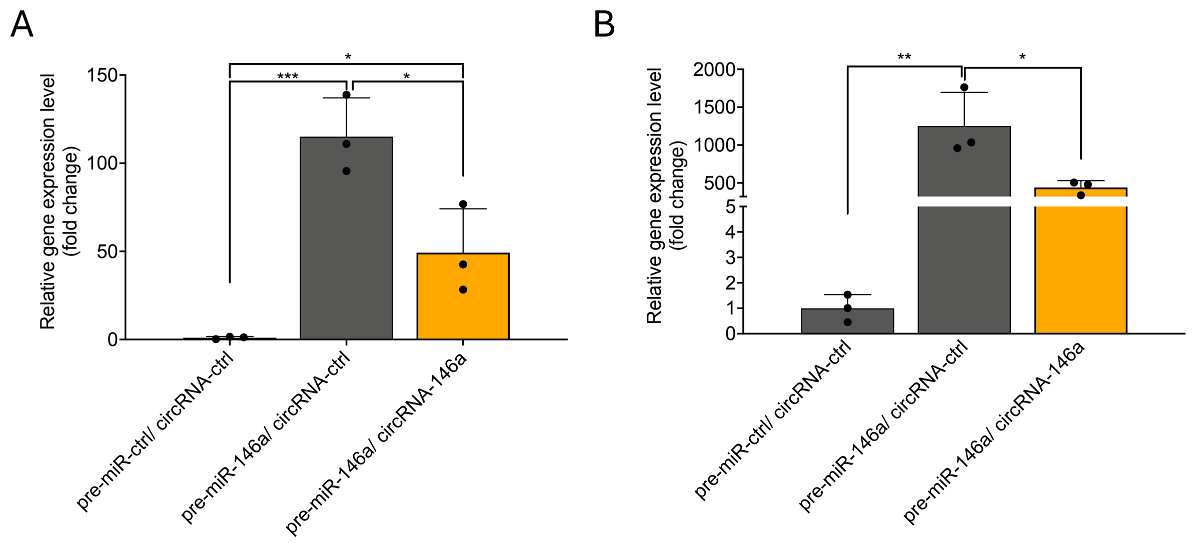

Supplementary Figure 6: Representative western blots of downstream target proteins following circRNA- or anti-miR treatment. A) Western blot analysis of target proteins PDCD4, PTEN, and SPRY1 of miRNA-21 after 2.5nM circRNA-21 or 50nM anti-miR-21 transfection. B) Western blot analysis of target proteins KLF4, TRAF6, and NRAS of miRNA-21 after 2.5nM circRNA-146a or 50nM anti-miR-146a transfection.

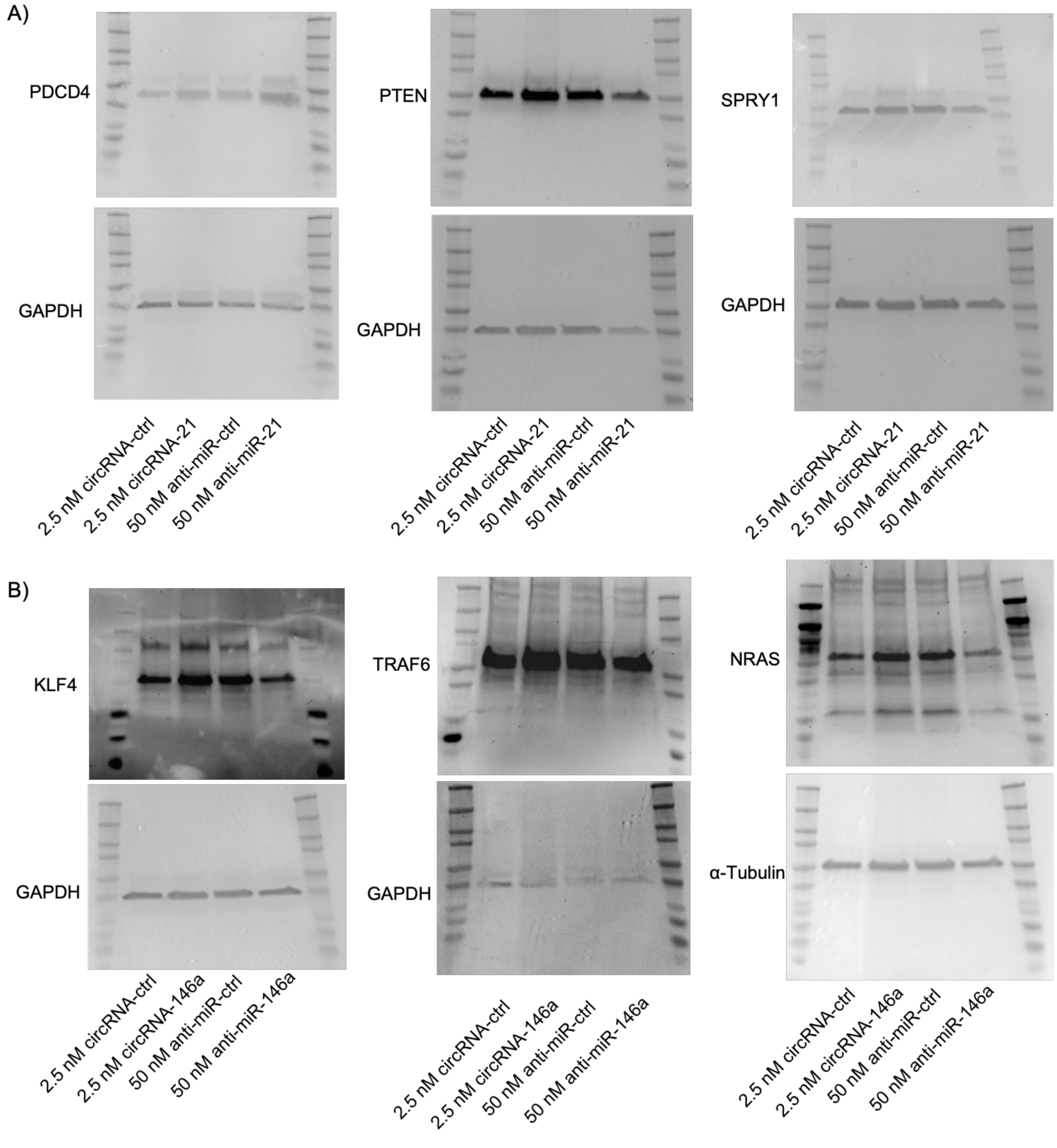

Supplementary Figure 7: Exemplary scratch wound assays (A) and live cell imaging (B) in VSMCs. Exemplary pictures at different time points after transfection of 2.5 nM circRNA-21, circRNA-146a or circRNA-control. Wound scratch area is framed in yellow. Counted cells are framed in purple.

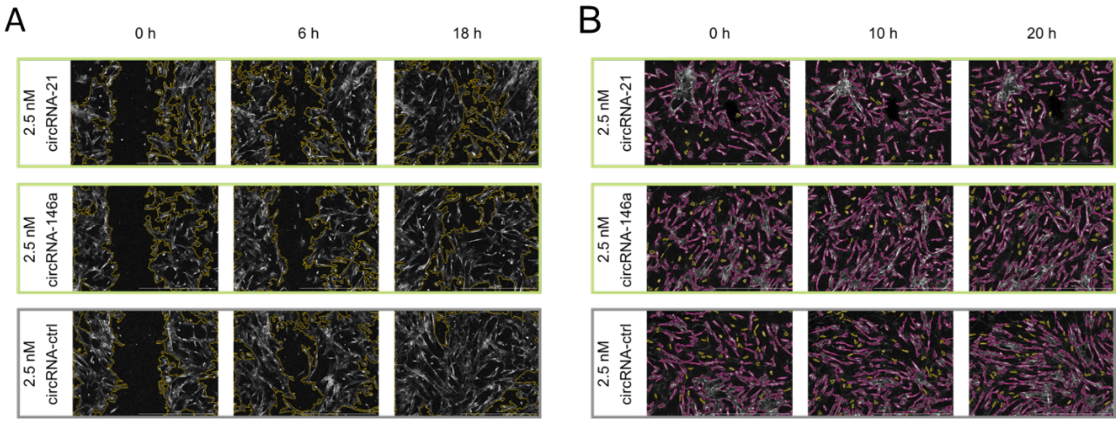

Supplementary Figure 8: CircRNAs influence miRNA-21 target regulation in HUVECs

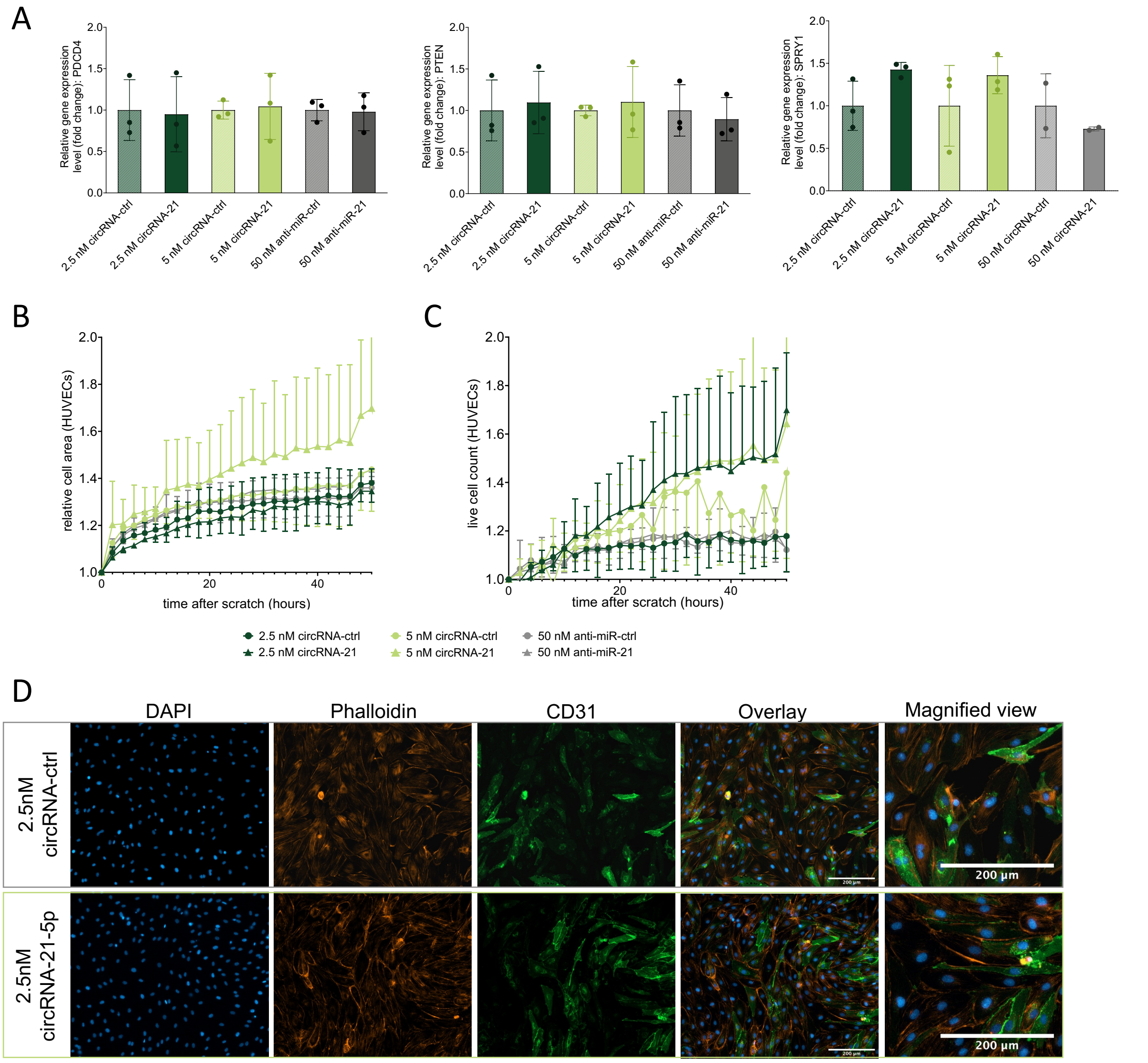

CircRNAs influence miR-21-5p target regulation and cellular function of HUVECs. A: (qRT)PCR analysis of miR-21-5p targets PDCD4 (left), PTEN (middle), and SPRY1 (right). Cells were transfected with either 2.5 nM, 5 nM circRNA, or 50 nM anti-miR in senescent HUVECs, n=3. B: The effect of circRNAs on cellular function is measured by migration capacity. Migration capacity was determined via scratch wound assay in HUVECs to inhibit miR-21-5p through circRNAs (n=4). C: The effect of circRNAs on cellular function measured by proliferation. Proliferation was determined by cell counting in life cell imaging of senescent HUVECs to inhibit miR-21-5p through circRNAs (n=4). D: Immunofluorescence staining of replicative senescent cells HUVECs transfected with either 2.5 nM circRNA-21-5p or 2.5 nM circRNA-control with staining for DAPI (blue), Phalloidin (red), and CD31 (green).

Supplementary Figure 9: CircRNAs influence miRNA-146a target regulation in HUVECs

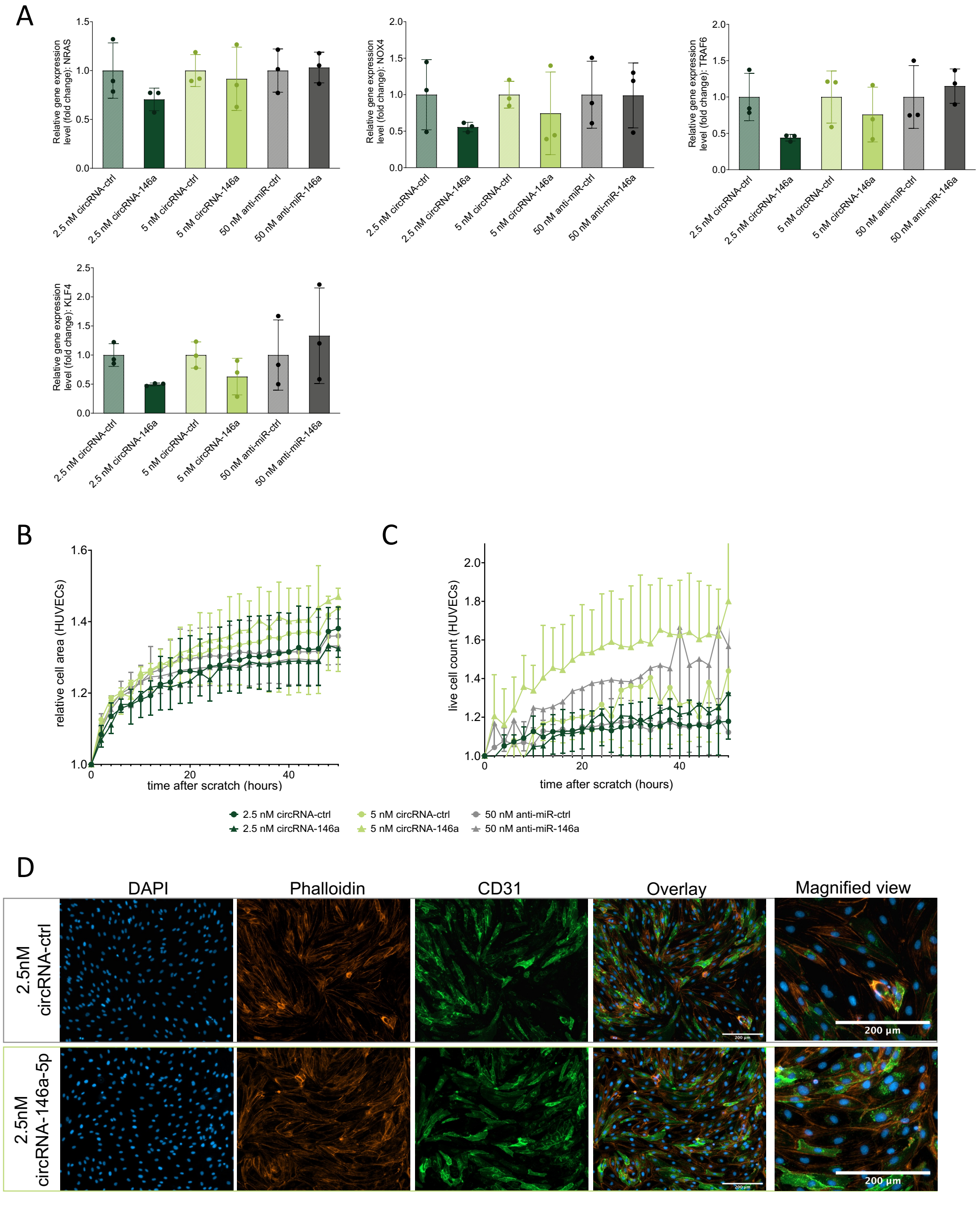

CircRNAs influence miR-146a-5p target regulation and cellular function of HUVECs. A: (qRT)PCR analysis of miR-146a-5p targets NRAS, NOX4, TRAF6, KLF4. Cells were transfected with either 2.5 nM, 5 nM circRNA, or 50 nM anti-miR in senescent HUVECs, n=3. B: The effect of circRNAs on cellular function is measured by migration capacity. Migration capacity was determined via scratch wound assay in HUVECs to inhibit miR-146a-5p through circRNAs (n=4). C: The effect of circRNAs on cellular function measured by proliferation. Proliferation was determined by cell counting in life cell imaging of senescent HUVECs to inhibit miR-146a-5p through circRNAs (n=4). D: Immunofluorescence staining of replicative senescent cells HUVECs transfected with either 2.5 nM circRNA-146a-5p or 2.5 nM circRNA-control with staining for DAPI (blue), Phalloidin (red), and CD31 (green).
